# CCT4 promotes tunneling nanotube formation

**DOI:** 10.1002/1873-3468.70190

**Published:** 2025-10-17

**Authors:** Miyu Enomoto, Akiko Asada, Taro Saito, Kanae Ando

**Affiliations:** ^1^ Department of Biological Sciences School of Science, Graduate School of Science, Tokyo Metropolitan University Japan

**Keywords:** CCT4, intercellular protein transport, microtubule dynamics, tunneling nanotube

## Abstract

Tunneling nanotubes (TNTs) are membranous tunnel‐like structures that mediate cell‐to‐cell communication, although the molecular mechanisms of TNT formation are not fully understood. T‐complex protein 1 subunit delta (CCT4) serves as a component of the chaperonin‐containing TCP1 complex (TRiC) and also functions as a monomer. Here, we report that monomeric CCT4 promotes TNT formation in mammalian cultured cells. The expression of GFP–CCT4 proteins, which are not incorporated into the chaperonin oligomer, induces the formation of nanotubes containing actin fibers and mitochondria. CCT4 proteins are transported intercellularly via these nanotubes. The expression of monomeric CCT4 enhances microtubule dynamics and increases tubulin‐containing TNTs. Our results suggest a newly identified function of monomeric CCT4 in TNT formation.

**Impact statement**

Tunneling nanotubes (TNTs) play critical roles in various physiological and pathological conditions. TNTs vary in their morphology and cytoskeleton. We found that cells expressing monomeric CCT4 generate many thick TNTs with tubulin. Our results suggest that CCT4 drives the formation of microtubule‐containing TNTs.

## Graphical abstract

Tunneling nanotubes (TNTs) are membranous tunnel‐like structures that transport molecules and organelles between cells. They vary in thickness, and thick nanotubes often contain microtubules in addition to actin fibers. We found that cells expressing monomeric CCT4 generate many thick TNTs with tubulin. Our results suggest that CCT4 drives the formation of microtubule‐containing tunneling nanotubes.

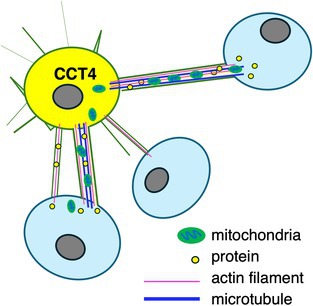

## Abbreviations

CCT4, chaperonin‐containing T‐complex protein 1 Subunit delta

EB1, end‐binding protein 1

F‐actin, filamentous actin

TNT, tunneling nanotube

TRiC, chaperonin‐containing TCP1 complex

Tunneling nanotubes (TNTs) are open membranous channels between connected cells, allowing direct intercellular communication [1, 2, 3]. TNTs have lengths in a broad range of 3–100 μm and widths ranging from 50 nm to 2 μm [4], contain filamentous actin (F‐actin) and sometimes microtubules, and transport small molecules such as calcium ions, macromolecules such as nucleic acids and proteins, and large cargos, including organelles such as vesicles, lysosomes, autophagosomes, and mitochondria, to mediate fast and specific responses between cells [1].

TNTs also play important roles in stress responses and disease pathogenesis [5, 6]. TNT‐mediated material transfers regulate cancer phenotypes such as invasiveness, metabolic plasticity, and therapy resistance [5]. In neurodegenerative diseases, including Parkinson's and Alzheimer's disease, TNTs transfer protein aggregates from neurons to microglia to protect neurons [7] or spread to other neurons via TNTs to propagate [8, 9, 10, 11]. TNT‐mediated mitochondrial transfer can protect stressed cells or boost cellular fitness [7, 12]. Despite their physiological and pathological importance, the molecular mechanisms of TNT formation are not fully understood.

CCT4 is a component of the chaperonin‐containing TCP1 complex (CCT/TRiC) [13]. TRiC is formed by eight subunits and assists in the folding of various proteins involved in the cytoskeleton, translation, protein degradation, and signaling [14]. Some CCT subunits also function as monomers, working independently of TRiC [15, 16, 17]. CCT4 has been shown to influence the cytoskeletal structure and regulate F‐actin organization independently of TRiC. In cultured cells, overexpression of a monomeric form of CCT4 induces actin‐based protrusions at the cell surface [15], which are caused by CCT4 interaction with p150^Glued^ [16]. In this study, we set out to investigate whether CCT4 is also involved in TNT formation.

## Materials and methods

### Chemicals

Fluorescent‐labeled phalloidin St. Louis, MO, USA (Phalloidin‐Atto 647N, Sigma, catalog # 65906) and MitoTracker Green FM Waltham, MA, USA (Thermo Fisher Scientific, catalog # M7514) were purchased.

### Plasmids

GFP‐CCT4 and GFP‐CCT2 [15] were provided by Dr. Julie Grantham (University of Gothenburg). mCherry‐CCT4 was constructed using NEB Builder HiFi DNA Assembly Ipswich, MA, USA (New England Biolabs, catalog # E2621S). CCT4 was amplified by PrimeSTAR Max DNA polymerase (TAKARA BIO) using primers, 5′‐CTAGCCAGCTCGTCCATGCCCGGGAAGCTACGTTCTCCGG‐3′ and 5′‐GATGTGGTAAATACTCGATAACTAGATAACTGATCATAATC‐3′ and assembled into pmCherry‐C1. EGFP‐tubulin‐6 was purchased from Addgene (catalog # 56450) (Rizzo et al., Cold Spring Harb Protoc. 2019). GFP‐Lifeact was purchased from Addgene (catalog # 58470). mCherry‐EB1 was constructed by subcloning mouse EB1 from mouse brain cDNAs into pmCherry‐N1A by NEB builder.

### Cell culture and transfection

Neuro‐2a (RRID: CVCL_0470) was obtained from Dr. Masao Takeuchi at the Institute for Fermentation, Osaka, Japan. Neuro‐2a cells were cultured in minimum essential medium (MEM) supplemented with 1% nonessential amino acids, 10% fetal bovine serum (FBS), 100 U·mL^−1^ penicillin, and 0.1 mg·mL^−1^ streptomycin. HeLa (RRID:CVCL_0030) was purchased from the RIKEN BioResource Center, Japan (RCB0007). HeLa cells were cultured in D‐MEM (High Glucose) with L‐Glutamine and Phenol Red with 10% fetal bovine serum (FBS), 100 U·mL^−1^ penicillin, and 0.1 mg·mL^−1^ streptomycin. These cell lines are microplasma‐free and have been authenticated in the past three years by morphology check by microscope and growth curve analysis.

Cells were transfected with plasmids using Lipofectamine 2000 and Lipofectamine 3000 Carlsbad, California, USA (Invitrogen, catalog # L3000015 and 11 668 019) according to the manufacturer's protocol. Neuro2A cells stably expressing the fluorescent protein DsRed were established by transfecting DsRed plasmids using Lipofectamine 2000, followed by selection with medium containing 500 μg·mL^−1^ G418 (WAKO, catalog # 078–05961) for two weeks, replacing the medium every 2–3 days. Surviving clones were isolated, expanded, and screened for stable expression of DsRed using Keyence microscope BZ‐X700 Osaka, Japan (Keyence).

### Imaging fixed cells

HeLa and Neuro 2a cells were plated on coverslips and transfected. Cells were fixed with 4% PFA in PBS for 20 min at 37 °C, permeabilized with 0.1% Triton X‐100 and stained with fluorescent‐labeled phalloidin or DAPI. Samples were observed using a confocal microscope Zeiss LSM710 (Zeiss), and the obtained results were analyzed by ImageJ [18].

### Live cell imaging

Neuro 2a cells were plated on a noncoated glass‐bottom dish, transfected with indicated plasmids, and observed under a confocal microscope Zeiss LSM710 (Zeiss). Results were analyzed by ImageJ (NIH) [19].

### Statistics

Statistical analyses were carried out with microsoft excel (Microsoft), python (Python 3), and graphpad prism (GraphPad). Differences were assessed using the Student's *t*‐test, chi‐squared test, or one‐way ANOVA followed by Tukey's HSD. *P* values < 0.05 were considered statistically significant.

## Results

### Monomeric CCT4 promotes nanotubes that connect neighboring cells

**Fig. 1:**
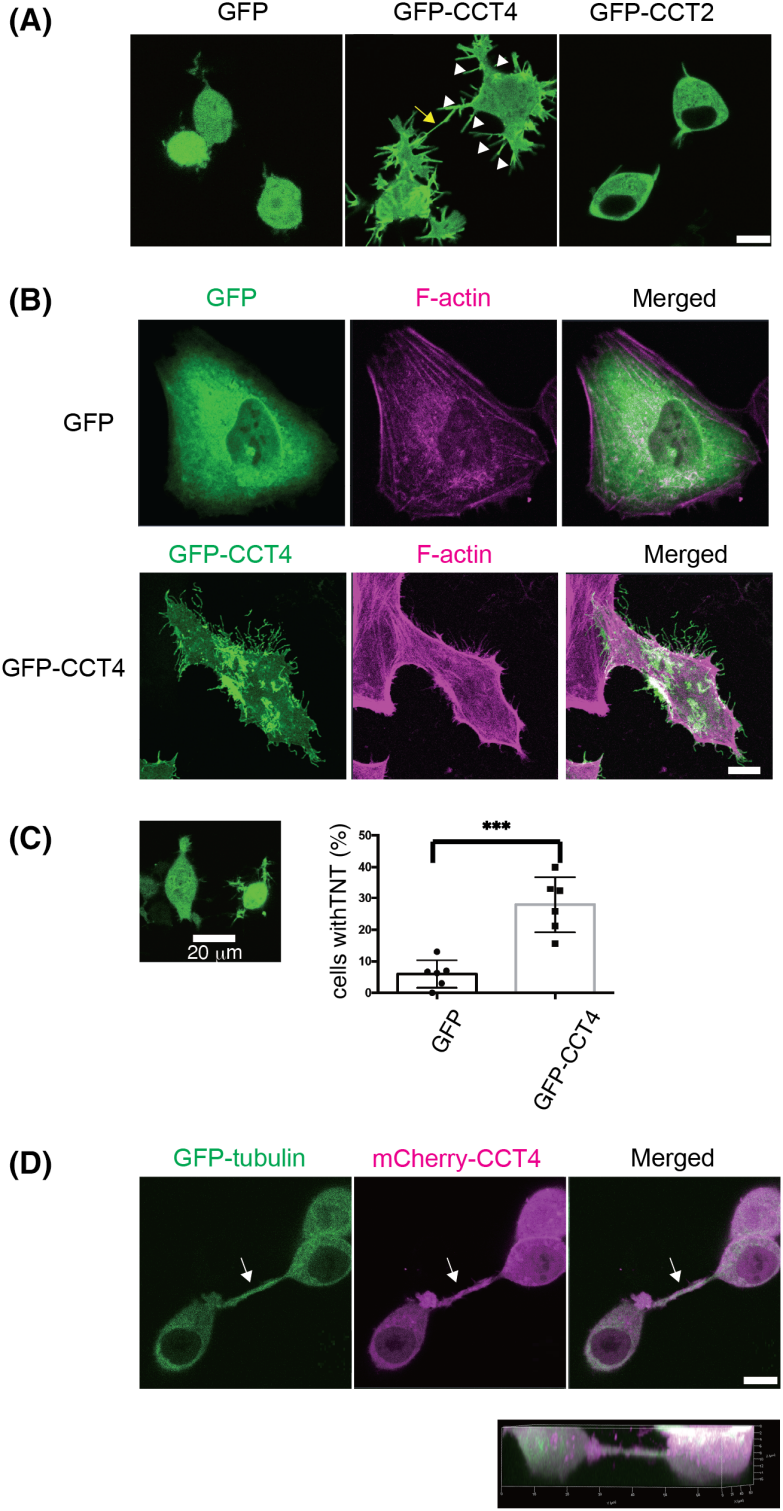
GFP‐CCT4 induces tunneling nanotube formation in cultured cells. (A) Live cell imaging of Neuro2A cells transfected with GFP, GFP‐CCT4, and GFP‐CCT2. GFP‐CCT4 expression induces the formation of protrusions (arrowheads) and nanotubes connecting two cells (arrow). Scale bar, 10 μm. (B) HeLa cells transfected with GFP and GFP‐CCT4. Cells were fixed and stained with phalloidin (magenta) and DAPI (blue) to visualize actin fibers and nuclei. GFP‐CCT4 expression induces formation of protrusions. Scale bar, 10 μm. (C) GFP‐CCT4‐transfected cells develop nanotubes more often than GFP‐transfected cells. Cells with nanotubes with the neighboring cells more than 20 μm away were scored. Scale bar, 20 μm. Thirty cells in each of 6 independent wells were analyzed. Mean ± SD, ***P < 0.001; Student's *t*‐test. (D) Nanotubes extended from CCT4‐expressing cells are floating above coverslips. Live imaging of Neuro2A cells transfected with GFP‐Tubulin (green) and mCherry‐CCT4 (magenta). A full‐focus image of Z‐stuck (top) and a 3D‐reconstructed image (bottom). The arrow indicates the floating tube. scale bar, 10 μm.

To analyze the function of monomeric CCT4 proteins, we used CCT4 tagged with GFP in its N terminus (GFP‐CCT4), which is not incorporated into the chaperonin oligomer [15]. In Neuro2A cells and HeLa cells, GFP‐CCT4 was distributed to the plasma membrane (Fig. 1A,B), as reported with B16F1 cells previously [15]. Cells with GFP‐CCT4 expression had numerous protrusions, as previously reported [15]. In addition, those cells have nanotube‐like structures, which are thicker and longer than protrusions and connect neighboring cells (Fig. 1A,B, protrusions (arrow heads) and a tube connecting cells (arrow)). These nanotubes are often more than 1 μm in thickness and connect cells apart more than 20 μm. Formation of many protrusions and nanotube‐like structures was not observed with cells transfected with GFP alone (Fig. 1A, GFP) or those transfected with chaperonin‐containing TCP1 complex subunit 2 (CCT2) (Fig. 1A, GFP‐CCT2). We analyzed the percentage of cells with nanotubes for Neuro2A cells expressing GFP and those expressing GFP‐CCT4 and found that cells with GFP‐CCT4 expression have nanotubes significantly more often: about 5% of GFP‐expressing cells and about 30% of GFP‐CCT4‐expressing cells have nanotubes (Fig. 1C). Nanotubes thicker than 1 μm contain tubulin: 22 out of 22 nanotubes from three independent experiments were tubulin‐positive.

GFP‐CCT4 expression has been reported to induce retraction fibers and filopodia‐like protrusions [15], while the formation of nanotubes has not been reported previously. Retraction fibers and filopodia‐like protrusions are attached to the substrate, while TNTs are hovering over the substrate. We found that the nanotubes from GFP‐CCT4‐expressing cells are floating above coverslips (Fig. 1D), indicating that these structures are not retraction fibers or filopodia.

### Nanotubes induced by GFP‐CCT4 contain F‐actin and mitochondria

**Fig. 2:**
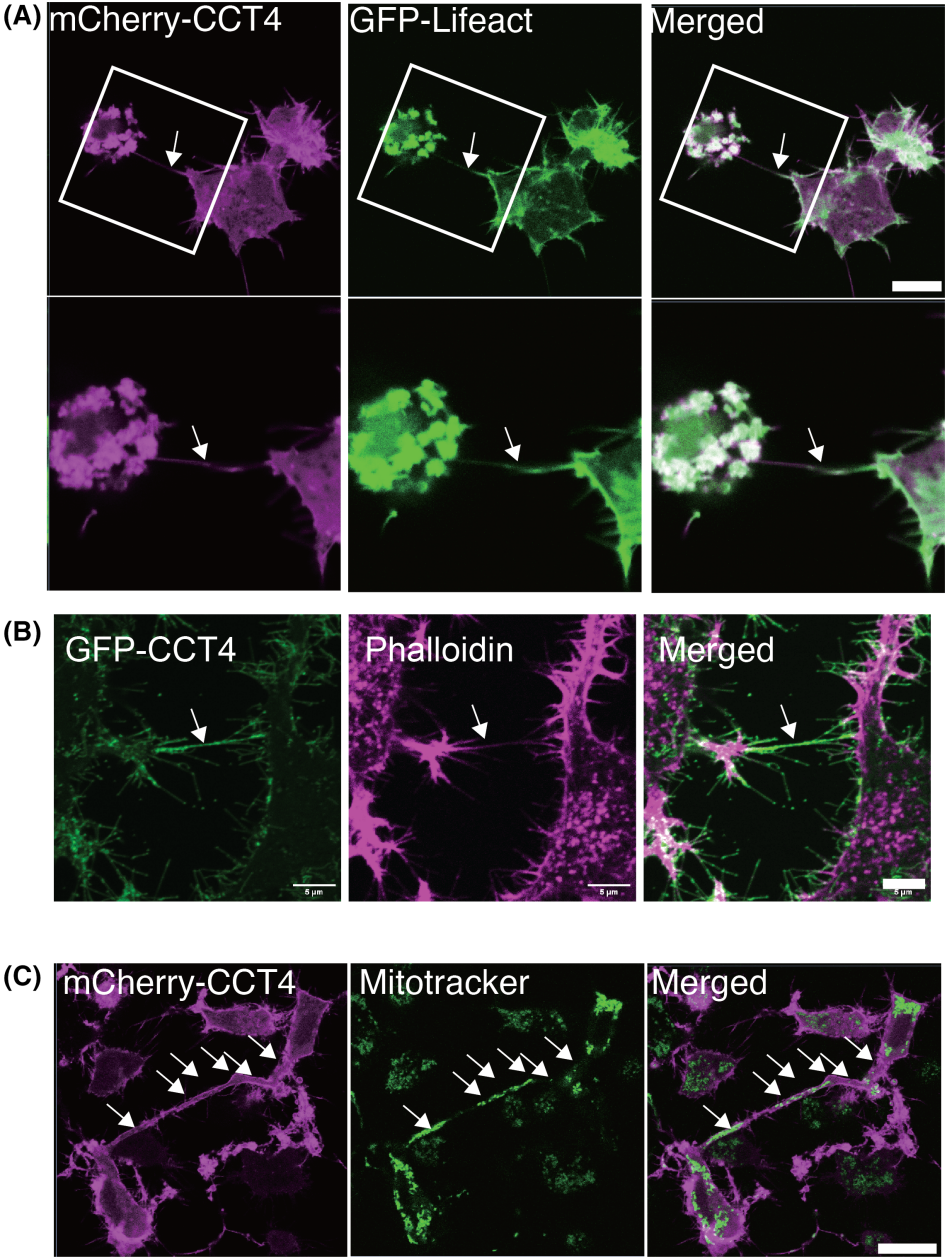
GFP‐CCT4 expression induces the formation of nanotubes containing F‐actin and tubulin puncta. (A) Live‐imaging of Neuro2A transfected with mCherry‐CCT4 (magenta) and GFP‐Lifeact (green). Arrows indicate a nanotube connecting two cells. Scale bar, 10 μm. (B) Neuro2A was transfected with GFP‐CCT4 (green) and stained with phalloidin (magenta). Arrows indicate the nanotube connecting two cells. Scale bar, 5 μm. (C) Live imaging of Neuro2A cells transfected with mCherry‐CCT4 (magenta) was stained with Mitotracker (green). Arrows indicate mitochondria. Scale bar, 20 μm.

TNTs contain actin fibers and microtubules, and organelles such as mitochondria are transported on microtubules in TNTs [1]. We asked whether the nanotubes of cells with GFP‐CCT4 expression have these features. Live imaging of cells expressing GFP‐LifeAct and staining of fixed cells with fluorescent‐labeled phalloidin showed that CCT4‐induced nanotubes connecting two cells contain actin fibers (Fig. 2A,B). GFP staining with a mitochondrial dye, mitotracker, revealed that these nanotubes contain mitochondria (Fig. 2C).

### Nanotubes induced by GFP‐CCT4 connect neighboring cells

**Fig. 3:**
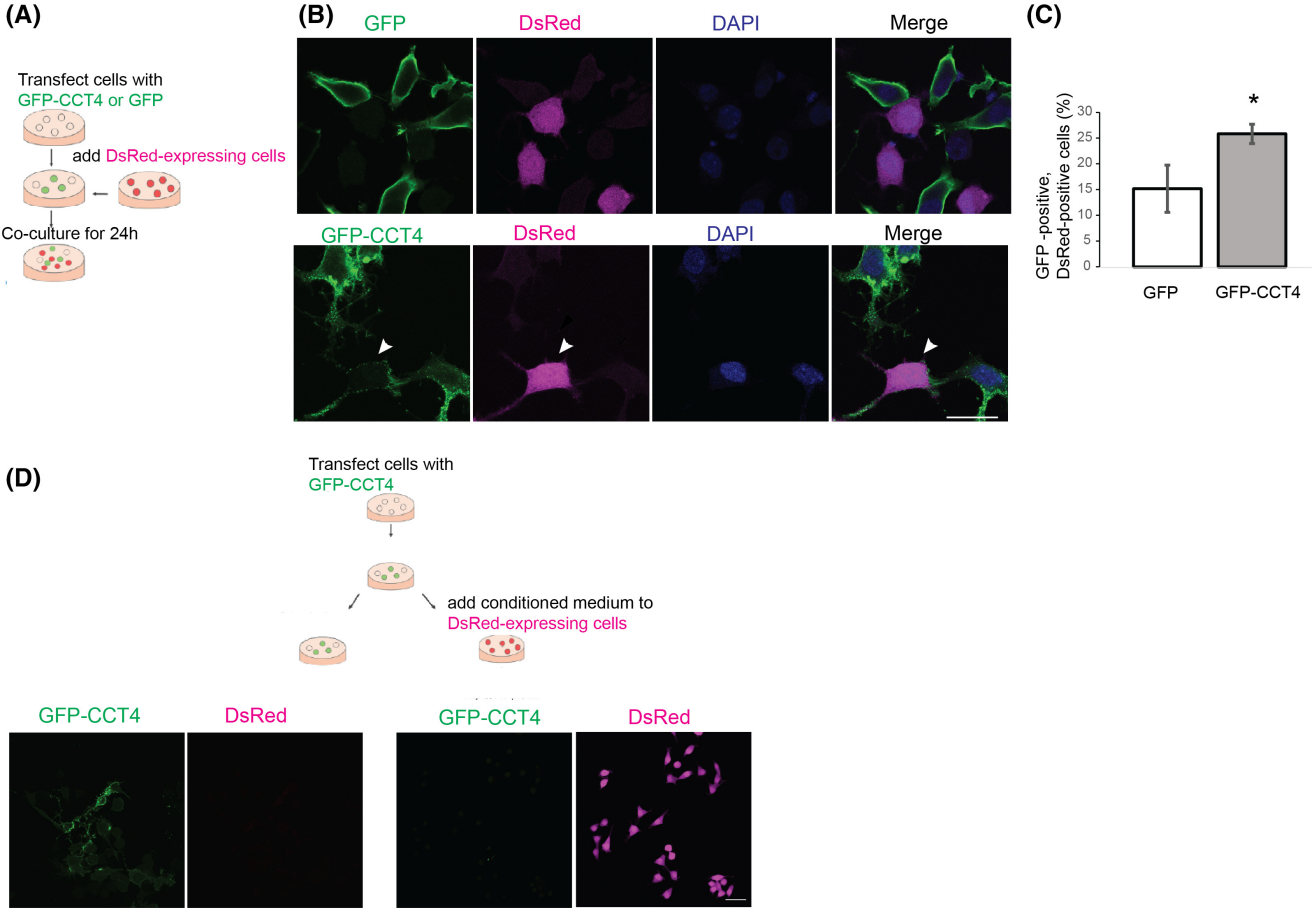
GFP‐CCT4 is transferred to neighboring cells via nanotubes. GFP‐CCT4 spreads to neighboring cells that are connected with tunneling nanotubes (TNTs). (A) Experimental scheme. (B) Neuro2A cells transfected with GFP or those transfected with GFP‐CCT4 were mixed with DsRed stable transfectants. Cells were counterstained with DAPI (blue). The arrowhead indicates a DsRed‐positive cell with GFP‐CCT4. (C) The number of cells positive for GFP, those for DsRed, and those for both GFP and DsRed were counted (top) and expressed as the percentage of GFP‐positive cells (bottom). Mean ± SE, *n* = 3, *P < 0.05; Student's t‐test. (D) DsRed‐expressing Neuro2A cells were incubated with a conditioned medium of cells transfected with GFP‐CCT4 for 24 h. Left: Cells transfected with GFP‐CCT4. Right: DsRed‐expressing Neuro2A incubated with conditioned medium. No GFP‐positive cells were observed. scale bar, 10 μm.

If nanotubes from cells expressing GFP‐CCT4 connect neighboring cells and are open‐ended, they would transfer proteins. We asked whether GFP‐CCT4 proteins are transferred intercellularly using a coculture experiment. Cells transfected with GFP‐CCT4, GFP‐CCT2, or GFP were mixed with cells that stably express DsRed. After 24 h of coculture, GFP signals in DsRed‐positive cells were analyzed (Fig. 3A). In the wells of cells transfected with GFP‐CCT4, we found some DsRed‐cells connected with GFP‐CCT4‐expressing cells show GFP signal, suggesting that GFP‐CCT4 proteins were transferred (Fig. 3B, arrowheads). DsRed cells with GFP signal were more frequently observed with the coculture of GFP‐CCT4 expressing cells than with the coculture of GFP‐expressing cells (Fig. 3C), suggesting that GFP‐CCT4 can facilitate the intracellular transfer of proteins.

Proteins can be intercellularly transported via the extracellular medium. We asked whether CCT4 is transferred to neighboring cells via the conditioned medium. Cells expressing DsRed were cultured for 24 h in the conditioned medium collected from cells transfected with GFP‐CCT4 (Fig. 3D). There were no DsRed cells that were positive for GFP (Fig. 3D). These results suggest that TNT formed in GFP‐CCT4‐expressing cells is open‐ended and transfers proteins between cells.

### Monomeric CCT4 enhances tubulin polymerization in TNTs

**Fig. 4:**
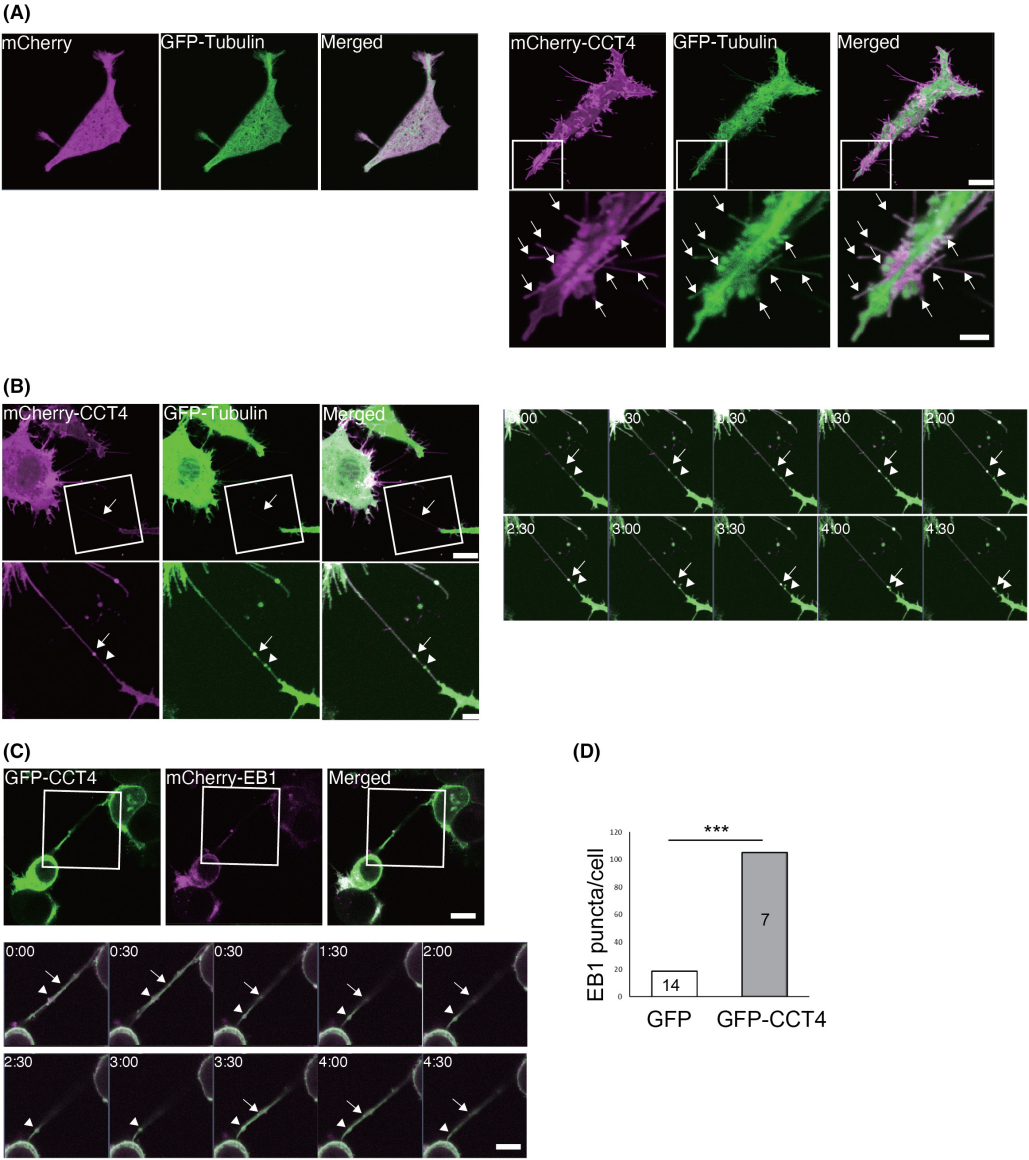
Overexpression of CCT4 enhances microtubule dynamics. (A) Neuro2A cells transfected with mCherry (magenta) and GFP‐αtubulin (green) (Left) and mCherry‐CCT4 (magenta) and GFP‐α tubulin (Green) (Right). Blow‐up images are shown in the bottom panels. Arrows indicate co‐localization of mCherry‐CCT4 and GFP‐α tubulin. scale bars, 10 μm (top) and 5 μm (bottom). (B) Neuro2A cells were transfected with mCherry‐CCT4 (magenta) and GFP‐α tubulin (green) and imaged every 30 s for 5 min. GFP‐ α tubulin formed puncta in tunneling nanotubes (TNTs) (arrow and arrowhead), which were moving at approximately 80 nm·s^−1^. The arrow indicates tubulin puncta colocalized with CCT4. Scale bar, 10 μm. (C) Neuro 2A cells were transfected with GFP‐CCT4 and mCherry‐EB1. Blow‐up images of the TNT are shown at the bottom. The arrow and arrowhead indicate CCT4 puncta in TNT, and the arrow indicates CCT4 puncta overlapping with EB1. scale bars, 10 μm (top) and 5 μm (bottom). (D) GFP‐CCT4 increased the number of EB1 puncta. Three different areas containing about five cells each were imaged, and the number of EB1 puncta was counted. The numbers in the bars indicate the number of cells analyzed. ***P < 0.001; Chi‐square test.

To visualize the remodeling of the cytoskeleton in these structures, we cotransfected mCherry‐CCT4 and GFP‐tubulin. GFP‐tubulin distributes to TNTs extending from cells expressing GFP‐CCT4 (Fig. 4A, Movies S1 and S2). Interestingly, GFP‐tubulin puncta are moving from cells with GFP‐CCT4 toward the neighboring cells connected with TNTs at the speed of approximately 80 nm·min^−1^ (Fig. 4B). To observe the microtubule elongation, we co‐expressed end‐binding protein 1 (EB1) fused with mCherry, which labels the microtubule growing end [20]. Live imaging of Neuro2A cells co‐expressing GFP‐CCT4 and mCherry‐EB1 revealed that the expression of GFP‐CCT4 increased the number of EB1 puncta (Fig. 4C,D, Movie S3). EB1 puncta were also observed in TNTs, and CCT4 was observed as puncta nearby or sometimes overlapping with tubulin puncta or EB1 puncta (arrows in Fig. 4B,C, Movie S4). These results suggest that monomeric CCT4 regulates microtubule organization in the cytosol and TNTs.

## Discussion

In this study, we found that monomeric CCT4 induces TNT formation (Figs 1, 2, 3). TNTs have been reported in various conditions and cellular contexts, and they are variable in their morphology and cytoskeleton content [1]. While some TNTs contain F‐actin alone as the cytoskeleton, some contain both F‐actin and microtubules [1]. Expression of monomeric CCT4 increased the number of thick TNTs containing tubulin (Fig. 1) and CCT4‐induced TNTs contain growing microtubules (Fig. 4), suggesting that CCT4 promotes the formation of nanotubes containing microtubules.

Although TRiC is known to mediate the folding of cytoskeletal proteins, including tubulins [21, 22], the role of monomeric CCT4 in microtubule polymerization has not been reported previously. Connectome analyses revealed that CCT4 proteins interact with tubulins [18, 20, 23, 24, 25, 26] and proteins involved in microtubule remodeling such as tubulin tyrosine ligase‐like family member 3 [18, 23, 24], histone deacetylase 6 [27], histone deacetylase 11 [26], cyclin‐dependent kinase 5 [28, 29, 30], and cilia and flagella‐associated protein 52 [31]. Although these analyses do not distinguish proteins interacting with the TRiC complex from those with monomeric CCT4, some of these proteins may interact with monomeric CCT4 and regulate microtubule dynamics in TNTs. It has been reported that the expression of GFP‐CCT4 in cultured mammalian cells induces retraction fiber formation [15], and this phenotype is caused by interactions between CCT4 and p150^Glued^ [16]. Interestingly, an isoform of p150^Glued^ expressed primarily in neurons interacts with tubulin dimers and microtubules and functions as an anticatastrophic factor [32]. Morphological changes caused by GFP‐CCT4 include increased membrane curvature and lipid composition changes, such as increased phosphatidylethanolamine lipid species [33]. Such lipid composition alterations may increase membrane fluidity and might contribute to TNT formation. Further studies on proteins interacting with monomeric CCT4 in TNTs may provide insights into mechanisms underlying their morphological and functional variation.

CCT4 has also been linked to neurological diseases: a mutation in CCT4/5 subunits causes sensory neuropathy [34], and CCT4 expression is upregulated in AD [35]. Functions of CCT4 in TNT formations may also contribute to understanding the pathogenesis of these disorders.

## Conflicts of interest

The authors declare no conflict of interest.

## Author contributions

Conceptualization: AA, ME, and KA, Methodology: AA, ME, and KA, Formal analysis: AA, ME, and KA, Investigation: AA, ME, and KA, Writing – original draft preparation: ME and KA, Writing – review and editing: AA, TS, and KA, Supervision: AA, TS, and KA, Project administration: KA, Funding acquisition: KA.

## Supporting information


**Movie S1.** GFP‐ αtubulin (green) in Neuro2A cells expressing mCherry.

**Movie S2.** GFP‐ αtubulin (green) in Neuro2A cells expressing mCherry‐CCT4.

**Movie S3.** GFP‐ αtubulin (green) and mCherry‐CCT4 (magenta) in the nanotube (Fig. 4B).

**Movie S4.** mCherry‐EB1 in Neuro 2A cells expressing GFP‐CCT4 (Fig. 4C).

## Data Availability

The datasets used in this study are available from the corresponding author upon request.

## References

[1] Cordero Cervantes D and Zurzolo C (2021) Peering into tunneling nanotubes‐the path forward. EMBO J 40, e105789. 33646572 10.15252/embj.2020105789 PMC8047439

[2] Rustom A , Saffrich R , Markovic I , Walther P and Gerdes HH (2004) Nanotubular highways for intercellular organelle transport. Science 303, 1007–1010. 14963329 10.1126/science.1093133

[3] Sartori‐Rupp A , Cordero Cervantes D , Pepe A , Gousset K , Delage E , Corroyer‐Dulmont S , Schmitt C , Krijnse‐Locker J and Zurzolo C (2019) Correlative cryo‐electron microscopy reveals the structure of TNTs in neuronal cells. Nat Commun 10, 342. 30664666 10.1038/s41467-018-08178-7 PMC6341166

[4] Saha T , Dash C , Jayabalan R , Khiste S , Kulkarni A , Kurmi K , Mondal J , Majumder PK , Bardia A , Jang HL *et al*. (2022) Intercellular nanotubes mediate mitochondrial trafficking between cancer and immune cells. Nat Nanotechnol 17, 98–106. 34795441 10.1038/s41565-021-01000-4 PMC10071558

[5] Pinto G , Brou C and Zurzolo C (2020) Tunneling nanotubes: the fuel of tumor progression? Trends Cancer 6, 874–888. 32471688 10.1016/j.trecan.2020.04.012

[6] Chastagner P , Loria F , Vargas JY , Tois J , Diamond IM , Okafo G , Brou C and Zurzolo C (2020) Fate and propagation of endogenously formed tau aggregates in neuronal cells. EMBO Mol Med 12, e12025. 33179866 10.15252/emmm.202012025 PMC7721367

[7] Scheiblich H , Eikens F , Wischhof L , Opitz S , Jungling K , Cserep C , Schmidt SV , Lambertz J , Bellande T , Posfai B *et al*. (2024) Microglia rescue neurons from aggregate‐induced neuronal dysfunction and death through tunneling nanotubes. Neuron 112, 3106–3125. 39059388 10.1016/j.neuron.2024.06.029

[8] Abounit S , Bousset L , Loria F , Zhu S , de Chaumont F , Pieri L , Olivo‐Marin JC , Melki R and Zurzolo C (2016) Tunneling nanotubes spread fibrillar alpha‐synuclein by intercellular trafficking of lysosomes. EMBO J 35, 2120–2138. 27550960 10.15252/embj.201593411 PMC5048354

[9] Dilsizoglu Senol A , Samarani M , Syan S , Guardia CM , Nonaka T , Liv N , Latour‐Lambert P , Hasegawa M , Klumperman J , Bonifacino JS *et al*. (2021) alpha‐synuclein fibrils subvert lysosome structure and function for the propagation of protein misfolding between cells through tunneling nanotubes. PLoS Biol 19, e3001287. 34283825 10.1371/journal.pbio.3001287 PMC8291706

[10] Chakraborty R , Nonaka T , Hasegawa M and Zurzolo C (2023) Tunnelling nanotubes between neuronal and microglial cells allow bi‐directional transfer of alpha‐synuclein and mitochondria. Cell Death Dis 14, 329. 37202391 10.1038/s41419-023-05835-8 PMC10195781

[11] Tardivel M , Begard S , Bousset L , Dujardin S , Coens A , Melki R , Buee L and Colin M (2016) Tunneling nanotube (TNT)‐mediated neuron‐to neuron transfer of pathological tau protein assemblies. Acta Neuropathol Commun 4, 117. 27809932 10.1186/s40478-016-0386-4 PMC5096005

[12] Baldwin JG , Heuser‐Loy C , Saha T , Schelker RC , Slavkovic‐Lukic D , Strieder N , Hernandez‐Lopez I , Rana N , Barden M , Mastrogiovanni F *et al*. (2024) Intercellular nanotube‐mediated mitochondrial transfer enhances T cell metabolic fitness and antitumor efficacy, cell. Cell 187, 6614–6630. 39276774 10.1016/j.cell.2024.08.029 PMC11623344

[13] Yam AY , Xia Y , Lin HT , Burlingame A , Gerstein M and Frydman J (2008) Defining the TRiC/CCT interactome links chaperonin function to stabilization of newly made proteins with complex topologies. Nat Struct Mol Biol 15, 1255–1262. 19011634 10.1038/nsmb.1515 PMC2658641

[14] Gestaut D , Zhao Y , Park J , Ma B , Leitner A , Collier M , Pintilie G , Roh SH , Chiu W and Frydman J (2023) Structural visualization of the tubulin folding pathway directed by human chaperonin TRiC/CCT. Cell 186, 2038. 37116473 10.1016/j.cell.2023.04.004

[15] Spiess M , Echbarthi M , Svanstrom A , Karlsson R and Grantham J (2015) Over‐expression analysis of all eight subunits of the molecular chaperone CCT in mammalian cells reveals a novel function for CCTdelta. J Mol Biol 427, 2757–2764. 26101841 10.1016/j.jmb.2015.06.007

[16] Echbarthi M , Vallin J and Grantham J (2018) Interactions between monomeric CCTdelta and p150(glued): a novel function for CCTdelta at the cell periphery distinct from the protein folding activity of the molecular chaperone CCT. Exp Cell Res 370, 137–149. 29913154 10.1016/j.yexcr.2018.06.018

[17] Brackley KI and Grantham J (2010) Subunits of the chaperonin CCT interact with F‐actin and influence cell shape and cytoskeletal assembly. Exp Cell Res 316, 543–553. 19913534 10.1016/j.yexcr.2009.11.003

[18] Huttlin EL , Bruckner RJ , Navarrete‐Perea J , Cannon JR , Baltier K , Gebreab F , Gygi MP , Thornock A , Zarraga G , Tam S *et al*. (2021) Dual proteome‐scale networks reveal cell‐specific remodeling of the human interactome. Cell 184, 3022–3040. 33961781 10.1016/j.cell.2021.04.011 PMC8165030

[19] Schneider CA , Rasband WS and Eliceiri KW (2012) NIH image to ImageJ: 25 years of image analysis. Nat Methods 9, 671–675. 22930834 10.1038/nmeth.2089 PMC5554542

[20] Go CD , Knight JDR , Rajasekharan A , Rathod B , Hesketh GG , Abe KT , Youn JY , Samavarchi‐Tehrani P , Zhang H , Zhu LY *et al*. (2021) A proximity‐dependent biotinylation map of a human cell. Nature 595, 120–124. 34079125 10.1038/s41586-021-03592-2

[21] Geissler S , Siegers K and Schiebel E (1998) A novel protein complex promoting formation of functional alpha‐ and gamma‐tubulin. EMBO J 17, 952–966. 9463374 10.1093/emboj/17.4.952 PMC1170445

[22] Vainberg IE , Lewis SA , Rommelaere H , Ampe C , Vandekerckhove J , Klein HL and Cowan NJ (1998) Prefoldin, a chaperone that delivers unfolded proteins to cytosolic chaperonin. Cell 93, 863–873. 9630229 10.1016/s0092-8674(00)81446-4

[23] Huttlin EL , Bruckner RJ , Paulo JA , Cannon JR , Ting L , Baltier K , Colby G , Gebreab F , Gygi MP , Parzen H *et al*. (2017) Architecture of the human interactome defines protein communities and disease networks. Nature 545, 505–509. 28514442 10.1038/nature22366 PMC5531611

[24] Cho NH , Cheveralls KC , Brunner AD , Kim K , Michaelis AC , Raghavan P , Kobayashi H , Savy L , Li JY , Canaj H *et al*. (2022) OpenCell: endogenous tagging for the cartography of human cellular organization. Science 375, eabi6983. 35271311 10.1126/science.abi6983 PMC9119736

[25] Huttlin EL , Ting L , Bruckner RJ , Gebreab F , Gygi MP , Szpyt J , Tam S , Zarraga G , Colby G , Baltier K *et al*. (2015) The BioPlex network: a systematic exploration of the human interactome. Cell 162, 425–440. 26186194 10.1016/j.cell.2015.06.043 PMC4617211

[26] Liu X , Salokas K , Tamene F , Jiu Y , Weldatsadik RG , Ohman T and Varjosalo M (2018) An AP‐MS‐ and BioID‐compatible MAC‐tag enables comprehensive mapping of protein interactions and subcellular localizations. Nat Commun 9, 1188. 29568061 10.1038/s41467-018-03523-2 PMC5864832

[27] Xu X , Ding P , Shi L , Wu G and Ma X (2022) LukS‐PV inhibits hepatocellular carcinoma cells migration by downregulating HDAC6 expression. BMC Cancer 22, 630. 35676659 10.1186/s12885-022-09680-4 PMC9175482

[28] Buljan M , Ciuffa R , van Drogen A , Vichalkovski A , Mehnert M , Rosenberger G , Lee S , Varjosalo M , Pernas LE , Spegg V *et al*. (2020) Kinase Interaction Network Expands Functional and Disease Roles of Human Kinases. Mol Cell 79, 504–520. 32707033 10.1016/j.molcel.2020.07.001 PMC7427327

[29] So J , Pasculescu A , Dai AY , Williton K , James A , Nguyen V , Creixell P , Schoof EM , Sinclair J , Barrios‐Rodiles M *et al*. (2015) Integrative analysis of kinase networks in TRAIL‐induced apoptosis provides a source of potential targets for combination therapy. Sci Signal 8, rs3. 25852190 10.1126/scisignal.2005700

[30] Varjosalo M , Sacco R , Stukalov A , van Drogen A , Planyavsky M , Hauri S , Aebersold R , Bennett KL , Colinge J , Gstaiger M *et al*. (2013) Interlaboratory reproducibility of large‐scale human protein‐complex analysis by standardized AP‐MS. Nat Methods 10, 307–314. 23455922 10.1038/nmeth.2400

[31] Silva FP , Hamamoto R , Nakamura Y and Furukawa Y (2005) WDRPUH, a novel WD‐repeat‐containing protein, is highly expressed in human hepatocellular carcinoma and involved in cell proliferation. Neoplasia 7, 348–355. 15967112 10.1593/neo.04544 PMC1501145

[32] Lazarus JE , Moughamian AJ , Tokito MK and Holzbaur EL (2013) Dynactin subunit p150(glued) is a neuron‐specific anti‐catastrophe factor. PLoS Biol 11, e1001611. 23874158 10.1371/journal.pbio.1001611 PMC3712912

[33] Fletcher JS , Samfors S , Vallin J , Svanstrom A and Grantham J (2021) Correlated fluorescence microscopy and multi‐ion beam secondary ion mass spectrometry imaging reveals phosphatidylethanolamine increases in the membrane of cancer cells over‐expressing the molecular chaperone subunit CCTdelta. Anal Bioanal Chem 413, 445–453. 33130974 10.1007/s00216-020-03013-9 PMC7806562

[34] Lee MJ , Stephenson DA , Groves MJ , Sweeney MG , Davis MB , An SF , Houlden H , Salih MA , Timmerman V , de Jonghe P *et al*. (2003) Hereditary sensory neuropathy is caused by a mutation in the delta subunit of the cytosolic chaperonin‐containing t‐complex peptide‐1 (Cct4) gene. Hum Mol Genet 12, 1917–1925. 12874111 10.1093/hmg/ddg198

[35] Guttula SV , Allam A and Gumpeny RS (2012) Analyzing microarray data of Alzheimer's using cluster analysis to identify the biomarker genes. Int J Alzheimers Dis 2012, 649456. 22482075 10.1155/2012/649456 PMC3296213

